# Disease-Associated Regulation of Non-Coding RNAs by Resveratrol: Molecular Insights and Therapeutic Applications

**DOI:** 10.3389/fcell.2022.894305

**Published:** 2022-07-13

**Authors:** Roberta Giordo, Zena Wehbe, Anna Maria Posadino, Gian Luca Erre, Ali H. Eid, Arduino A. Mangoni, Gianfranco Pintus

**Affiliations:** ^1^ College of Medicine, Mohammed Bin Rashid University of Medicine and Health Sciences, Dubai, United Arab Emirates; ^2^ Vascular Biology Research Centre, Molecular and Clinical Research Institute, University of London, London, United Kingdom; ^3^ Department of Biomedical Sciences, University of Sassari, Sassari, Italy; ^4^ Rheumatology Unit, Department of Clinical and Experimental Medicine, University Hospital (AOUSS) and University of Sassari, Sassari, Italy; ^5^ Department of Basic Medical Sciences, College of Medicine, Q.U. Health. Qatar University, Doha, Qatar; ^6^ Discipline of Clinical Pharmacology, College of Medicine and Public Health, Flinders University, Adelaide, SA, Australia; ^7^ Department of Clinical Pharmacology, Flinders Medical Centre, Adelaide, SA, Australia; ^8^ Department of Medical Laboratory Sciences, College of Health Sciences and Sharjah Institute for Medical Research, University of Sharjah, Sharjah, United Arab Emirates

**Keywords:** non-coding RNAs, ncRNAs, resveratrol, atherosclerosis, diabetes, obesity, cancer

## Abstract

There have been significant advances, particularly over the last 20 years, in the identification of non-coding RNAs (ncRNAs) and their pathophysiological role in a wide range of disease states, particularly cancer and other chronic conditions characterized by excess inflammation and oxidative stress such as atherosclerosis, diabetes, obesity, multiple sclerosis, osteoporosis, liver and lung fibrosis. Such discoveries have potential therapeutic implications as a better understanding of the molecular mechanisms underpinning the effects of ncRNAs on critical homeostatic control mechanisms and biochemical pathways might lead to the identification of novel druggable targets. In this context, increasing evidence suggests that several natural compounds can target ncRNAs at different levels and, consequently, influence processes involved in the onset and progression of disease states. The natural phenol resveratrol has been extensively studied for therapeutic purposes in view of its established anti-inflammatory and antioxidant effects, particularly in disease states such as cancer and cardiovascular disease that are associated with human aging. However, increasing *in vitro* and *in vivo* evidence also suggests that resveratrol can directly target various ncRNAs and that this mediates, at least in part, its potential therapeutic effects. This review critically appraises the available evidence regarding the resveratrol-mediated modulation of different ncRNAs in a wide range of disease states characterized by a pro-inflammatory state and oxidative stress, the potential therapeutic applications, and future research directions.

## Introduction

Over the last two decades of the post-genomic era, it has become evident that only about 1.5% of the human genome corresponds to protein-coding sequences whereas the remaining 98.5% is non-coding (Lander et al., 2001). Large consortia, such as the Encyclopedia of DNA Elements (ENCODE) and its official Gencode gene catalog (www.gencodegenes.org) (Derrien et al., 2012), have been instrumental in the expansion of the human gene catalog with the addition of thousands of validated and annotated non-coding genes. These initiatives have also facilitated the generation of evidence supporting the participation of non-coding genes, once merely regarded as “junk”, in the regulation of the activity of protein-coding genes (Palazzo and Lee, 2015). Specifically, the discovery of several types of non-coding RNAs (ncRNAs) and their involvement in a wide range of homeostatic processes has revolutionized our understanding of the pathogenesis of disease states (Costa, 2005). NcRNAs are a heterogenous group of RNA molecules with different biological function, biogenesis, structure, and length. Based on the arbitrary length of 200 nucleotides (nt), ncRNAs are divided into two classes: small (sncRNAs) (<200 nt) and long (>200 nt) (lncRNAs) (Zhang P. et al., 2019). lncRNAs are widely expressed in various tissues and cells lines and their spatial and temporal expression profiles as well as their specific interactions with DNA, RNA and proteins, reflect distinct functions (Statello et al., 2021). Indeed, lncRNAs participate in regulating cell cycle and cell differentiation, physiological processes such as development, aging, and immunity, and pathological processes such as cancer, cardiovascular disease and diabetes (Leung and Natarajan, 2018; Fu P.-F. et al., 2019; Dehaini et al., 2019; Wehbe et al., 2019; Zhang C. et al., 2020; Giordo et al., 2021a; Statello et al., 2021). lncRNAs regulate gene expression at both transcriptional and post-transcriptional level. At the transcriptional level, they can affect target genes either in cis, regulating the expression of neighboring genes, or in trans, away from their loci, interacting with chromatin at several thousand different locations across multiple chromosomes (Zhang X. et al., 2019). Another category of ncRNAs (a subtype of the sncRNAs class), the microRNAs (miRNAs), have also gained significant popularity over the last two decades and revolutionized our knowledge of the mechanisms regulating health and disease states. miRNAs have a length of approximately 22 nt and typically bind to the 3′ untranslated region (3′UTR) of mRNAs, inducing mRNA degradation and translational repression, although miRNAs inducing mRNA translation have been also reported (Dragomir et al., 2018). miRNAs can target multiple mRNAs and, at the same time, mRNAs are targeted by multiple miRNAs (Peter, 2010; O'Brien et al., 2018). The complexity of these regulatory networks is further compounded by the involvement of lncRNAs as competing endogenous RNAs (ceRNAs), or miRNAs sponges, with the consequent reduction or inhibition of the regulatory effect of miRNAs on their mRNA target(s) (Tay et al., 2014; López-Urrutia et al., 2019). A wide range of biological processes is regulated through the crosstalk lncRNAs-miRNAs and the aberrant expression of miRNAs is associated with various pathological states. In addition, miRNAs can be packed into extracellular vesicles such as exosomes, serving as messengers that mediate cell-cell communication or function through intercellular signaling pathways. Emerging evidence, mainly in the context of cancer research, suggests that the expression of ncRNAs, as well as their downstream targets, can be modulated by the intake of specific nutrients and natural compounds (Ferrero et al., 2021; Scoditti et al., 2021). For instance, natural products such as curcumin, resveratrol, and epigallocatechin-3-gallate, have been shown to exhibit anti-cancer effects by modulating the expression and the function of several ncRNAs, with consequent inhibition of cancer cell growth, induction of apoptosis, and increased therapeutic efficacy of conventional cancer therapy (Zhang B. et al., 2020; Sabo et al., 2021). Within the above mentioned natural compounds, resveratrol (3, 5, 4′-trihydroxystilbene), a nonflavonoid polyphenol contained in various foods, e.g., grapes, apples, blueberries, cocoa and peanuts, has been extensively studied in view of its numerous biological effects (Galiniak et al., 2019). Such effects are mediated by multiple pathways and targets and include antioxidant, anti-inflammatory, neuroprotective, cardioprotective, immunomodulatory, anti-platelet, and anticancer properties (Koushki et al., 2018; Shaito et al., 2020; Giordo et al., 2021b). Moreover, resveratrol has been found to modulate several aspects of cancer biology, such as tumor cell growth, migration, invasion, and resistance/evasion to apoptosis, by controlling the expression of miRNAs and lncRNAs (Wang et al., 2019). However, the ability of resveratrol to modulate the expression profile of ncRNAs has been also reported in other pathophysiological states (Latruffe et al., 2015; Zhu et al., 2020b; Shu et al., 2021). In this review, we critically appraise the available evidence regarding the resveratrol-mediated regulation of miRNAs and lncRNAs in various pathological conditions, particularly cancer, metabolic disorders, inflammation and ischemia.

## Insulin Resistance, Diabetes, and Obesity

The hormone insulin, secreted by pancreatic β-cells in response to high blood glucose, lowers serum glucose concentrations by stimulating glucose uptake into the cells (Fu et al., 2013). Insulin resistance, a condition associated with reduced cell sensitivity to insulin, predisposes to type 2 diabetes (T2D) (Cerf, 2013). Recent studies have shown that resveratrol and metformin (MET), a first-line drug for the management of type 2 diabetes (Giannarelli et al., 2003), exert glucose-lowering effects through similar mechanisms, i.e., reduced hepatic gluconeogenesis, inflammation and oxidative stress, mainly through the activation of NAD-dependent deacetylase SIRT1 and AMP-activated protein kinase (AMPK) (Dludla et al., 2020). Based on this finding, a study has sought to determine whether such effects are mediated by lncRNAs, performing a comparative analysis of liver tissue lncRNAs expression profiles by high-throughput sequencing from high-fat diet (HFD)-fed mice treated with resveratrol and MET (Shu et al., 2021). Both compounds attenuated liver insulin resistance by regulating lncRNAs, although MET exerted a more prominent effect. Moreover, both resveratrol and MET affected the PI3K/Akt signaling pathway through the NONMMUT034936.2 and G6PC target genes (Shu et al., 2021). By using the same experimental model (HFD-fed mice), another study identified mmu-miR-363-3p as the major miRNA involved in the beneficial effects of resveratrol on insulin resistance. Specifically, resveratrol improved insulin resistance by upregulating mmu-miR-363-3p via the PI3K-Akt pathway, and reducing the expression of FOXO1 and G6PC, the downstream effectors of the PI3K-Akt pathway (Shu et al., 2020). The modulation of miR-33a/b and miR-122 has also been proposed as a treatment strategy for insulin resistance and dyslipidemia associated with obesity and metabolic syndrome; miR-122 is strongly associated with the risk of developing metabolic syndrome and type 2 diabetes (Willeit et al., 2017) whereas the downregulation of hepatic miR-33 improves metabolic homeostasis and liver function (Price et al., 2021). Proanthocyanidins, a polyphenolic compounds extracted from grape seeds, have been shown to suppress these key miRNAs in the liver of healthy and obese rats (Baselga-Escudero et al., 2015). In addition, Baselga-Escudero et al. investigated the modulatory effects of resveratrol and epigallocatechin gallate, polyphenols that are instead mainly found in the grape skin, toward these miRNAs (Baselga-Escudero et al., 2013). Both resveratrol and epigallocatechin gallate modulated miR-33 and miR-122 concentrations by direct binding, and the effect on miRNA expression was structure-dependent. The specific binding of polyphenols to miRNAs represents a new post-transcriptional mechanism by which polyphenols can modulate metabolism (Baselga-Escudero et al., 2013). Finally, resveratrol treatment in diabetic mice upregulated miR-18a-5p, a promising therapeutic target for preventing and attenuating diabetic nephropathy (Xu et al., 2017). Insulin resistance is also closely associated with the metabolic syndrome, a cluster of risk factors specific for cardiovascular disease such as obesity, high blood pressure, and dyslipidemia (Roberts et al., 2013). In the context of obesity, the main adipogenic genes involved in weight gain are regulated by different mechanisms, including miRNAs (Brandao et al., 2021). Another reported beneficial effect of resveratrol is body-fat lowering through the combined suppression of adipogenesis and lipogenesis (Aguirre et al., 2014). To identify the mechanisms underpinning this effect, Eseberri et al. investigated the possible involvement of miRNAs in the regulation of adipogenic transcription factors, peroxisome proliferator-activated receptor *γ* (pparγ) and CCAAT enhancer-binding proteins *α* and *β* (cebpβ and cebpα), by resveratrol and its glucuronide metabolites (Eseberri et al., 2017). Among all selected miRNAs (validated and predicted) targeting the adipogenic transcription factors, only miR-155 was modified by resveratrol and its metabolites. Specifically, up-regulation of miR-155, with the consequent down-regulation of the expression of the cebpβ gene, was observed (Eseberri et al., 2017). Additionaly, a miRNA microarray analysis in perirenal adipose tissue disclosed that resveratrol treatment led to the overexpression of miR-539-5p in the white adipose tissue of rats, and consequent inhibition of *de novo* lipogenesis, providing additional evidence of the anti-obesity effect of this flavonoid (Gracia et al., 2016). See Table 1 for summary.

**TABLE 1:** Insulin resistance, diabetes and obesity.

Non-coding RNA	Non-coding RNA function	Pathology	Resveratrol effect	Final effect	References
mmu-miR-363-3p		Insulin resistance	↑miRNA	Insulin resistance Improvement via the PI3K-Akt pathway in HepG2 cells	Shu et al., (2020)
miR-33 miR-122	miRNAs associated with the risk for developing T2D	Insulin resistance	↓ miRNAs	Insulin resistance improvement in hepatic cells	Baselga-Escudero et al., (2013)
miR-18a-5p	Controls autophagy activity through ATM.	Diabetic nephropathy	↑miRNA	Diabetic nephropathy amelioration by increasing autophagy in mouse kidney tissues and podocytes cell line	Xu et al., (2017)
miR-155		Obesity	↑miRNA	Downregulation of the adipogenic transcription factor CEBP-β in 3T3-L1 preadipocytes	Eseberri et al., (2017)
miR-539-5p		Obesity	↑miRNA	Inhibition of *de-novo* lipogenesis in white adipose tissue	Gracia et al., (2016)

The function of miRNAs, in the column “miRNA, function” is referred to the specific pathology reported in the table.

ATM, Atactic telangiectasis mutation; CEBP-β, CCAAT enhancer-binding protein β; ↑, increased expression/activity; ↓, decreased expression/activity.

## Inflammatory States

Inflammation plays a critical pathophysiological role in a wide range of pathological states, including cancer, diabetes, obesity, and neurodegenerative diseases (Furman et al., 2019; Fois et al., 2020; Malhab et al., 2021). A significant body of research has provided strong evidence that resveratrol has anti-inflammatory properties by targeting transcription factors such as NF-kB and AP-1, and the gene COX-2 (Meng et al., 2021). The link between resveratrol and inflammation correlated-ncRNAs is still under investigation and the molecular mechanisms underpinning this interaction are yet to be identified. Resveratrol seems to modulate several miRNAs involved in inflammatory pathways, such as the proinflammatory miR-155, the anti-inflammatory miR-663, and the oncogenic miR-21 21 (Latruffe et al., 2015). A recent study has also reported the potential involvement of the lncRNA MALAT-1, a biomarker of inflammation (Wang et al., 2021). Treatment with resveratrol in rats with sepsis-induced kidney damage was associated with lower circulating concentrations of this lncRNA compared to untreated animals (Wang et al., 2021). Moreover, resveratrol reduced the intracellular concentrations of key pro-inflammatory cytokines, i.e., TNF-α, IL-1β and IL-6 (Wang et al., 2021). Based on previous studies reporting the participation of several MALAT1-sponged miRNAs, including miR-205 and miR204, in the pathogenesis of acute kidney injury (AKI) (Li et al., 2016; Chen et al., 2018), Wang et al. investigated the potential involvement of these miRNAs in their model of sepsis-induced AKI and the effects of resveratrol (Wang et al., 2021). Resveratrol downregulated miR-205 expression, suggesting that the inhibition of the lncRNA MALAT1/miR-205 axis by this flavonoid protects against sepsis-induced AKI (Wang et al., 2021). High-throughput RNA sequencing of chondrocytes treated with the pro-inflammatory factor IL-1beta, in presence or absence of resveratrol, revealed several differentially expressed lncRNAs, miRNAs, and mRNAs. (Yi et al., 2021). Moreover, combining bioinformatics methods with RT-PCR validation, a competitive endogenous RNA (ceRNA) network, involving the lncRNA LINC00654, miR-210-5p, and the Opioid Growth Factor Receptor Like 1 (OGFRL1), was identified (Yi et al., 2021). In this network, the mutual interaction between lncRNAs and miRNAs may influence cellular metabolic activity and functions as lncRNAs may competitively bind miRNAs, weakening the miRNA responses on the mRNA target and regulating genes at the post-transcriptional level (Yi et al., 2021). Resveratrol treatment upregulated LINC00654 and OGFRL1 and downregulated miR-210-5p in chondrocytes, suggesting that LINC00654 acts as ceRNA by competitively occupying the shared binding sequences of miR-210-5p in the OGFRL1 mRNA, ultimately affecting its gene expression (Yi et al., 2021). Another association between resveratrol and miRNA has been identified by Song et al. (Song et al., 2016) in human THP-1 macrophages. In this study, resveratrol decreased the inflammatory response through the modulation of miR-Let7A, a well-known tumor suppressor miRNA that modulates inflammation and apoptosis. In another study in mice with induced allergic asthma, resveratrol downregulated the expression of miR-34a, with consequent overexpression of FOXP3, a critically important transcription factor for the development and function of asthma-implicated regulatory T-cells (Tregs) (Alharris et al., 2018). Resveratrol may also be effective in models of acute lung damage, e.g., following exposure to staphylococcal enterotoxin B (SEB) (Alghetaa et al., 2018). In mice, Alghetaa et al. (Alghetaa et al., 2018) showed that 5-day resveratrol treatment in SEB-treated animals resulted in 100% survival compared to 0% survival in untreated animals. miRNA expression analysis in lung immune cells showed altered expression of different ncRNAs, particularly miR193-a, which was significantly activated in SEB-treated animals and down-regulated after resveratrol treatment. miR193-a seems to promote anti-inflammatory responses through activation of TGF-beta signaling and death receptor-6 apoptotic pathways (Alghetaa et al., 2018). The authors suggest that the SEB-activated lung injury can be prevented by resveratrol through the modulation of these anti-inflammatory miRNAs (Alghetaa et al., 2018). It is well known that resveratrol is a strong Sirtuins activator, above all SIRT1 that possesses anti-inflammatory activity (Borra et al., 2005; Moraes et al., 2020). SIRT1 seems to exert its effects through miRNAs, particularly miR-204, however the molecular mechanisms involved are still unknown (Li et al., 2015). Using lipopolysaccharide (LPS)-stimulated mice microglia cell lines, Li et al. (Li et al., 2015) demonstrated that SIRT1 overexpression and miR-204 inhibition counteracted LPS-induced inflammation similarly to resveratrol treatment. The latter promoted the activity of SIRT1 through miR-204. A significant number of studies have reported that the anticancer effects of resveratrol are strongly correlated with its anti-inflammatory activity (Ren et al., 2021). In a mice study on chemopreventive mechanisms of colitis-associated tumorigenesis, resveratrol treatment decreased markers of inflammation such as IL-6, tumor necrosis factor-alpha (protein levels), and COX-2 mRNA (mRNA levels) (Altamemi et al., 2014). Over 100 miRNAs appeared involved in these effects. Among them, miRNA-101b and miRNA-455 were significantly upregulated, suggesting that the interplay with different miRNAs may play an important role in the pleiotropic effects of resveratrol (Altamemi et al., 2014). See Table 2 for summary.

**TABLE 2:** Inflammation.

Non-coding RNA	Non-coding RNA function	Pathology	Resveratrol effect	Final effect	References
MALAT-1 (lncRNA)	Pro-inflammatory	Sepsis-induced inflammation	↓ lncRNA	Sepsis-induced AKI relief by suppressing the MALAT1/MiR-205 axis in rats	Wang et al., (2021)
miR-Let7A	Anti-inflammatory	Inflammation	↑miRNA	Anti-inflammatory response in THP-1 macrophage	Song et al., (2016)
miR-34a	Potentially inhibits the T-regulatory cells mediated immune response by targeting FOXP3	Allergic asthma and associated inflammation in the lungs	↓ miRNA	Attenuation of allergy/asthma symptoms via FOXP3 over expression in mice	Alharris et al., (2018)
miR193-a	Anti-inflammatory action	SEB-induced acute lung injury	↓ miRNA	Protection against SEB-mediated toxicity by triggering anti-inflammatory pathways in mice	Alghetaa et al., (2018)
miRNA-101b miRNA-455	Anti-inflammatory properties	Colitis-associated tumorigenesis	↑miRNAs	Mitigation of colitis-associated tumorigenesis in mice	Altamemi et al., (2014)

The function of miRNAs, in the column “miRNA, function” is referred to the specific pathology reported in the table.

AKI, acute kidney injury; SEB, Staphylococcal enterotoxin B; FOXP3, forkhead box P3; ↑, increased expression/activity; ↓, decreased expression/activity.

## Liver Fibrosis and Pulmonary Vascular Remodeling

Factors such as high alcohol consumption, viral infections and autoimmune diseases can lead to persistent inflammation and hepatotoxic effects (Suk and Kim, 2015). Subsequently, fibrosis or scarring of the liver may occur due to overactivation of hepatic stellate cells (HSCs) and excessive deposits of extracellular matrix proteins (Suk and Kim, 2015; Zhu et al., 2020a). The more extreme forms, i.e., cirrhosis, may culminate in fatal systemic inflammation and liver failure. Treatment of the disease mainly involve strategies that mitigate the triggering factors or, ultimately, liver transplant (Zhu et al., 2020a). In this context, resveratrol-elicited reduction of liver failure (LF) severity is mainly associated with its anti-inflammatory and antioxidative activity (Hessin et al., 2017; Liu et al., 2017). In a rat model of LF, the liver tissue of resveratrol treated animals demonstrated well defined hepatocytes, in contrast to the untreated group which displayed necrotic cells with abnormal features such as collagen fiber hyperplasia and pseudolobuli formation (Zhu et al., 2020a). Various concentrations of resveratrol were able to reduce liver cell death, normalize liver lobule structure and decrease excess collagen fiber deposits. In addition, resveratrol at various doses upregulated the expression of the autophagy proteins Beclin1 and Atg7, with consequent increase in autophagy in experimental models of LF (Zhu et al., 2020a). In LF, autophagy is often blocked by preventing full fusion of autophagosomes with lysosomes, resulting in increased liver damage (Zou et al., 2020). Autophagy is an important mechanism that facilitates elimination of misfolded and aggregated proteins and dysfunctional organelles and prevents excessive growth (Ravanan et al., 2017). Thus, a potentially significant therapeutic effect of resveratrol consists in the ability to induce protective autophagy in LF models, mitigating inflammation and excessive growth of HSC cells. In rat models of LF, animals treated with resveratrol at various doses demonstrated a dose-dependent increase in phosphatase and tensin homolog (PTEN), which is an important inducer of apoptosis in hepatic stellate cells in LF (HSCs) (Zhu et al., 2020a). Owing to its ability to inhibit downstream mitogenic PI3K/AKT, PTEN mitigates cell activation, thereby exerting a protective effect against LF (Zhu et al., 2020a). The ability of resveratrol to increase PTEN was partially attributed to a significant reduction in miR-20a expression in rat LF models. This miR is associated with LF and, incidentally, contains binding sites for PTEN. Binding to PTEN results in its reduced activation and thus diminished ability to activate cell autophagy in LF. Thus, resveratrol has the potential to slow the development of LF by suppressing miR-20a (Zhu et al., 2020a). Resveratrol has also demonstrated beneficial effects in pulmonary hypertension (PH), a progressive disease of the pulmonary arteries which ultimately results in pulmonary remodeling, vasoconstriction and increased pulmonary arterial pressure (Thenappan et al., 2018). Vascular remodeling, characterized by excessive proliferation of pulmonary artery smooth muscle cells (PAMCs), is a key feature of PH and a target for intervention (Tao et al., 2019). Interestingly, resveratrol was recently shown to induce a protective effect *in vivo* against PH (Liu et al., 2020). Specifically, in rat models of monocrotaline (MCT)-induced PH, treatment with resveratrol attenuated pulmonary vascular remodeling (Liu et al., 2020). This was associated with the increased expression of miR-638. *In vitro*, resveratrol was able to prevent PDGF-induced over-proliferation in pulmonary arterial smooth muscle cells (PASMCs) (Liu et al., 2020). Previous studies have highlighted the crucial role of NR4A3, a type of NR4A nuclear receptor superfamily, in stimulating PASMCs proliferation (Rodríguez-Calvo et al., 2013; Wang et al., 2015). NR4A3 was later shown to upregulate PASMC proliferation in a cyclin D1-dependent manner (Liu et al., 2020). *In vitro*, miR-638 mimics diminished PASMC proliferation, whereas anti-miR-638 resulted in enhanced cell proliferation, highlighting the role of this miR in halting proliferation (Liu et al., 2020). Luciferase reporter assay ultimately demonstrated the miR-638 directly targets NR4A3, suggesting that its anti-proliferative effect is mediated by the inhibition of NR4A3, thereby preventing downstream activation of cyclin D1 (Liu et al., 2020). Thus, by upregulating the expression of miR-638, resveratrol demonstrates a protective role in subverting pulmonary vascular remodeling related to excessive PASMC proliferation. See Table 3 for summary. See Table 3 for summary.

**TABLE 3:** Liver fibrosis, pulmonary vascular remodeling, low ambient cardiovascular disease, multiple sclerosis, ischemia, and osteoporosis.

Non-coding RNA	Non-coding RNA function	Pathology	Resveratrol effect	Final effect	References
miR-20a	Promote disease progression	Liver fibrosis (LF)	↓ miRNA	Autophagy induction and activation of miR-20a-mediated PTEN/PI3K/AKT signaling pathway to attenuate LF in rat liver	Zhu et al., (2020a)
miR-638	Anti-proliferative effect	Pulmonary hypertension	↑miRNA	Prevention of pulmonary vascular remodeling by regulating NR4A3/cyclin D1 pathway in rat	Liu et al., (2020)
miR-149-5p	Unknown	Ischemia	↑miRNA	Protection against ischemia by up-regulation of Sirt1/miR-149-5p signaling in rat brain	Teertam et al., (2020)
miR-20b	Anti-angiogenic	Ischaemic myocardium	↑miRNA	Anti-angiogenic effects through miR-20b/HIF-1α/VEGF axis in rat	Mukhopadhyay et al., (2012)
miR-328	Hypertophy-associated miRNA	Myocardial Hypertrophy	↓ miRNA	Attenuation of myocardial hypertrophy by inhibiting cardiomyocyte apoptosis in murine models of cold-induced cardiac hypertrophy	Yin et al., (2015)
miR-124	Neuroprotective effect	Multiple sclerosis	↑miRNA	Multiple sclerosis attenuation by altering the miR-124/Sphingosine Kinase 1 Axis in murine models of multiple sclerosis	Gandy et al., (2019)

The function of miRNAs, in the column “miRNA, function” is referred to the specific pathology reported in the table.

PTEN, phosphatase and tensin homolog; P13K, Phosphoinositide 3-kinase; NR4A3, Nuclear Receptor Subfamily 4 Group A Member 3; SIRT1, Sirtuin 1; HIF-1α, Hypoxia Inducible Factor 1 Subunit α; VEGF, Vascular Endothelial Growth Factor; ↑, increased expression/activity; ↓, decreased expression/activity.

## Low Ambient Cardiovascular Disease, Multiple Sclerosis, and Osteoporosis

Cardiovascular disease (CVD) is one of the three leading causes of death worldwide (Yin et al., 2015). There has been an increasing recognition that cold temperature is a risk factor of CVD, particularly as it leads to cardiac hypertrophy and hypertension (Sun, 2010; Yin et al., 2015). In murine models of cold-induced cardiac hypertrophy, resveratrol was shown to mitigate myocardial enlargement, damage caused by fibrosis, and myofibril disarray. Interestingly, heart function, assessed as ejection fraction (EF) using echocardiogram, was also improved with resveratrol. A resveratrol-mediated reduction of the hypertrophy-associated miR-328 was also observed (Li et al., 2014; Yin et al., 2015). miR-328 has been shown to induce cardiac hypertrophy in murine models. This was associated with reduced sarcoplasmic reticulum Ca^2+-^ATPase (SERCA2a) levels, a major transporter of Ca^2+^ from the cytosol back into the sarcoplasmic reticulum (Li et al., 2014). As a result, there was increased accumulation of intracellular Ca^2+^, resulting in elevated calcineurin and NFATc3 nuclear translocation. Activation of this pathway has been shown to regulate cardiac hypertrophy (Molkentin, 2004). Therefore, the suppression of miR-328 expression caused by resveratrol might prevent SERCA2Aa downregulation and, consequently, cardiac hypertrophy.

Protective effects of resveratrol have also been observed in murine models of multiple sclerosis, mainly through a reduction in central nervous system inflammation (Gandy et al., 2019). Interestingly, the miR profile of the encephalitogenic CD4^+^ T cells from these mice was altered, particularly those related to cellular proliferation. Resveratrol significantly increased miR-124 in these cells, thereby allowing inhibition of its target gene sphingosine kinase 1 (SK1) (Gandy et al., 2019). As SK1 is a lipid enzyme involved in activating signaling pathways leading to cell migration, proliferation, and inflammation, while also preventing apoptosis (Proia and Hla, 2015; Li et al., 2021), its inhibition by miR-124 might account for the neuroprotective effects of resveratrol in these studies. Resveratrol has also shown promising microRNA-mediated effects in models of osteoporosis, a disabling condition characterized by degradation of the bone microstructure and reduction in bone mass (Das and Crockett, 2013). Previously, ovariectomized rats were shown to benefit from treatment with resveratrol, as indicated by enhanced bone mineral density (BMD) of the epiphyses (Liu et al., 2005). In an experiment conducted by Guo et al*.*, female ovariectomized rats treated with resveratrol demonstrated increased BMD compared to controls (Guo et al., 2015). Increased concentrations of serum calcium, osteocalcin, and calcium/phosphate ratios, as well as a reduction in calcium urinary excretion, were also observed (Guo et al., 2015). Examination of the miR profile in the treatment group highlighted a significantly reduced expression of miR-338-3p. miR-338-3p knockdown in human osteoblast (HOB) cells resulted in increased expression of runt-related transcription factor 2 (RUNX2), confirming the TargetScan prediction that this miR can directly bind to and inhibit its expression (Guo et al., 2015). Inhibition of miR-338-3p expression resulted in increased cell proliferation and calcium deposition. These effects are attributed to the effect of RUNX2 on osteoblasts and odontoblasts (Miyazaki et al., 2008; Li et al., 2011). This transcription factor is involved in the signaling pathways associated with bone morphogenetic protein (BMP) and transforming growth factor β (TGF β), both of which contribute to osteoblast development and growth (Guo et al., 2015). Thus, stimulation of RUNX2 ultimately results in downstream proliferation and differentiation of HOB cells. Resveratrol treatment likely removed the miR-338-3p-mediated suppression of RUNX2, facilitating differentiation of the cells which direct bone growth (Guo et al., 2015). See Table 3 for summary.

## Ischemia

Ischemic stroke, resulting from vessel obstruction in the brain, represents the most common type of stroke worldwide (Meschia and Brott, 2018). Sirtuin 1 (SIRT1), a member of the nicotinamide adenine dinucleotide-dependent deacetylases, is widely expressed in various tissues and exerts protective effects against cellular stress, especially those associated with ischemia (Pantazi et al., 2013). Such effects include the attenuation of energy depletion, oxidative stress, and inflammation. SIRT1 was previously shown to deacetylate inducers of apoptosis, particularly the tumor-suppressor p53 (Vaziri et al., 2001). This is associated with a neuroprotective effect in the context of brain ischemia, likely by preventing apoptosis and the associated inflammation (Hernández-Jiménez et al., 2013). In a recent study in an animal model of ischemia-reperfusion injury (IRI) via middle cerebral artery occlusion (MCAO), pre-treatment with resveratrol exerted protective effects mediated by SIRT1 modulation (Teertam et al., 2020). Twenty-four hours after injury, there was a significant reduction in p53 and downstream caspase-3 activity (Teertam et al., 2020). This was likely a result of the increased miR-149-5p activity on SIRT1 stimulation. Thus, resveratrol modulation of the miR-149-5p/SIRT1 axis might exert neuroprotective effects against ischemia by preventing the apoptotic and inflammatory actions of p53 (Teertam et al., 2020).

In a related study, resveratrol, and its commercially available form, longevinex, were also investigated in animal models of cardiac ischemia/reperfusion (I/R) (Mukhopadhyay et al., 2012). Both treatments exerted anti-angiogenic effects in the heart, with significant reduction in infarct size, especially when resveratrol was used in combination with γ-tocotrienol. Importantly, this effect was attributed to miR-20b, a miR which is significantly down-regulated in the ischemic heart (Mukhopadhyay et al., 2012). Resveratrol treatment significantly reversed this decrease, once again especially when combination with γ-tocotrienol or given as longevinex. Concomitantly, there was a significant decrease, within the heart tissue, in the expression of hypoxia-inducible factor-1α (HIF-1α) and vascular endothelial growth factor (VEGF) (Mukhopadhyay et al., 2012), both of which modulate angiogenesis (Lee et al., 2019; Sousa Fialho et al., 2019). The reduction in these target proteins was attributed to the resveratrol-induced increase in miR-20b, as treatment with antagomir-miR-20b restored their pre-treatment concentrations (Mukhopadhyay et al., 2012). It is important to highlight that the strongest effect on miR-20b expression, and the consequent reduction in HIF-1α and VEGF, was achieved with longevinex, which contains the phenolic anti-oxidants, quercetin and ferulic acid in addition to resveratrol (Mukhopadhyay et al., 2012). The miR-20b/HIF-1α/VEGF axis represents another important signaling pathway through which resveratrol, especially in combination with other antioxidants, can exert therapeutic effects. See Table 3 for summary.

## Cancer

### Breast Cancer

A significant number of *in vitro* and *in vivo* studies have shown that resveratrol possesses anti-cancer properties through the modulation of key processes such as cell growth, invasion, migration and metastasis (Varoni et al., 2016), as well as the stimulation of various anti-inflammatory, antioxidant, anti-apoptotic and anti-angiogenic mechanisms (Trapp et al., 2010; Varoni et al., 2016). Resveratrol has also shown adjuvant anti-cancer effects by enhancing the sensitivity of cancer cells to chemotherapeutics, consequently overcoming drug resistance (Ko et al., 2017). Recent studies have shown that resveratrol modulates the expression of important oncogenic drivers and tumor suppressors, including miRNAs and lncRNAs (Varoni et al., 2016; Anastasiadou et al., 2018). Breast cancer is the most diagnosed female cancer worldwide and the most common type of cancer in women in developing countries (Gabriel and Domchek, 2010; Bray et al., 2018). The effects of resveratrol on breast cancer are controversial (Bartolacci et al., 2018) due to the fact that, as a phytoestrogen, it possesses both estrogenic and anti-estrogenic activities in ERɑ-positive breast cancer (Bhat et al., 2001). Moreover, the effects of resveratrol are highly dependent of the administration protocol, e.g., dose, frequency, and route (Pasciu et al., 2010; Giordo et al., 2013; Posadino et al., 2015; Bartolacci et al., 2018; Posadino et al., 2019; Shaito et al., 2020). By using a combination of secondary mining of published data and experimental validations (i.e., the mRNA expression profile from the Gene Expression Omnibus database and a miRNA expression profile previously published (Venkatadri et al., 2016)), along with the bioinformatics tools, STITCH, miRDB, and *de novo* motifs (used to predict the transcription factors motifs for differentially expressed genes), a recent bioinformatics study investigated the transcription factors–miRNA–mRNA coregulatory network involved in the inhibition of breast cancer cell proliferation by resveratrol. (Zhou et al., 2022). This integrative investigation highlights as resveratrol inhibit human breast cancer cells (MCF-7) proliferation by modulating the expression of genes, transcription factors, and miRNAs involved in cell cycle and apoptosis, such as E2F2, JUN, FOS, BRCA1, CDK1, CDKN1A, TNF, and hsa-miR34a-5p. Moreover, the combined assessment of STITCH and miRDB databases revealed that the inhibition of MCF-7 proliferation resulted from a dual action, i.e., activation of TF cell cycle inhibitors and inhibition of antiapoptosis-associated miRNAs (Zhou et al., 2022). An additional anti-cancer effect of resveratrol consists in the increased sensitivity of breast cancer cells to the conventional anti-cancer agent adriamycin. A recent study (Zhang W. et al., 2019) suggested that resveratrol may upregulate miR-122-5p, consequently causing cell-cycle arrest of adriamycin-resistant breast cancer cells by targeting apoptosis and cell cycle key regulators such as B-cell lymphoma 2 (Bcl-2) and cyclin-dependent kinases (CDKs) (Zhang W. et al., 2019). Tumor-suppressive miRNAs modulation is another mechanism used by resveratrol to control breast cancer cell proliferation (Venkatadri et al., 2016; Otsuka et al., 2018). For instance, according to Otsuka et al. (Otsuka et al., 2018), by activating the p53 pathway, resveratrol induces the expression of tumor-suppressive miR-34a, miR-424, and miR-503; this, in turn, suppresses the splicing regulator heterogeneous nuclear ribonucleoprotein A1 (HNRNPA1), which is associated with oncogenesis and tumor progression and is generally up-regulated in several cancer types in addition to breast cancer, e.g., colorectal cancer, lung cancer, and glioma (Park et al., 2016; Kim et al., 2017). This tumor suppressor miRNAs-associated resveratrol antiproliferative effect has been also reported by Venkatadri et al. (Venkatadri et al., 2016). In this study, miR-125b-5p, miR-200c-3p, miR-409-3p, miR-122-5p and miR-542-3p mediated the decreased expression of anti-apoptotic proteins, including Bcl-2 and X-linked inhibitor of apoptosis (XIAP) and CDKs proteins specific for G-phase arrest (Venkatadri et al., 2016). An elegant *in vivo* study highlighted that resveratrol may differentially influence the expression of DNA methyltransferase 3b (DNMT3b) and miRNAs in tumor tissue vs. normal tissue in a dose-dependent manner (Qin et al., 2014). Specifically, eighty-nine estradiol-(E2)-dependent mammary carcinoma female rats received 21-day treatment with estradiol plus high- or low-dose resveratrol. High doses caused a reduction in DNMT3b, the predominant methyltransferase in breast tumorigenesis, in tumor tissue and a concomitant increase in normal tissue (Qin et al., 2014). In addition, high doses increased, >two-fold, miR21, -129, -204, and -489 in tumor, and caused their reduction, by 10–50%, in normal tissue compared to untreated animals. By contrast, low doses increased the expression of miR10a, a tumor suppressor, and miR10b, associated with metastasis and advanced disease, in tumor tissue (Qin et al., 2014). As emphasized by the authors (Qin et al., 2014), the dose-dependent effect of resveratrol on mammary carcinogenesis warrants further investigations to identify the optimal dose in terms of risk/benefit ratio. This issue is particularly relevant in view of the challenges associated with the inherent poor solubility and bioavailability of resveratrol (Shaito et al., 2020). See summary in Table 4.

**TABLE 4:** Cancer

Non-coding RNA	Non-coding RNA function	Pathology	Resveratrol effect	Final effect	References
miR-122-5p	Tumor suppressor	Breast cancer (BC)	↑miRNA	↓ of key anti-apoptotic (Bcl2) and cell cycle arrest proteins (CDKs) in adriamycin-resistant BC cells	Zhang et al., (2019b)
miR-34a, miR-424, miR-503	Tumor suppressors	Breast cancer (BC)	↑ all miRNAs	↓ tumor-associated factor A1 (HNRNPA1) in MCF-7 and MDA-MB-231 breast cancer cells	Otsuka et al., (2018)
miR-125b-5p, miR-200c-3p, miR-409-3p, miR-122-5p miR-542-3p	Tumor suppressors	Breast cancer (BC)	↑ all miRNAs	↓ anti-apoptotic proteins, Bcl2 and XIAP in MCF-7 and MDA-MB-231 breast cancer cells	Venkatadri et al., (2016)
miR-34a	Tumor suppressor	Ovarian cancer (OC)	↑miRNA	↓ anti-apoptotic proteins Bcl2 in OC cells	Yao et al., (2021)
PCAT29 (lncRNA)	Decreases both proliferation and migration of PC cells	Prostate cancer (PC)	↑ lncRNA	Inhibition of signaling pathway, IL-6/STAT3/miR-21, that mediate PCAT29 suppression DU145, LNCaP, and RWPE-1 PC cells lines	Al Aameri et al., (2017)
miR-17 family	Oncogenic	Prostate cancer (PC)	↓ miRNAs	Restoration of tumor suppressor gene PTEN in DU145 and 22Rv1 PC cells lines	Dhar et al., (2015)
DIO3OS (lncRNA)	Promotes EMT	Benign prostatic hyperplasia (BPH)	↑ lncRNA	Reversion of EMT in benign prostatic hyperplasia epithelial cells	Chen et al., (2021)
miR-96	Unknown	Colorectal cancer (CRC)	↑miRNA	Inhibition of KRAS in genetically engineered mice	Saud et al., (2014)
CCAT1 CRNDE, HOTAIR PCAT1 PVT1 SNHG16	LncRNAs associated with colorectal cancer	Colorectal cancer (CRC)	↓ lncRNAs	Inhibition of cancer cells proliferation by repressing expression of LncRNAs in CRC cell line HT-29	Cesmeli et al., (2022)
miR-200 family	Tumor mediators	Pancreatic cancer (PACA)	↓ miRNAs	Inhibition of tumor cells migration, invasion and EMT in PACA cell line AsPC-1	Fu et al., (2019a)
NEAT1 (lncRNA)	Tumor inducer	Myeloma	↓ lncRNA	Reversion of the negative effect of NEAT1 through the Wnt/β-catenin signaling pathway in multiple myeloma (MM) cells	Geng et al., (2018)
miR-221	Oncogenic	Melanoma	↓ miRNAs	Suppression of melanoma by inhibiting NF-κB/miR-221 in melanoma cell lines A375 and MV3	Wu and Cui, (2017)
AK001796	Oncogenic	Lung cancer	↓ lncRNA	inhibition of cancer cell growth in lung cancer cells A549	Yang et al., (2015)
miRNA-520h	Oncogenic	Lung cancer	↓ miRNA	Inhibition of transcriptional factor FOXC2 in lung cancer cells	Yu et al., (2013)

The function of miRNAs in the column “miRNA function” is referred to the specific pathology reported in the table.

Bcl-2, B-cell leukemia/lymphoma-2 (apoptosis suppressor); CDKs, cyclin-dependent kinases (cell cycle regulators); HNRNPA1, Heterogeneous nuclear ribonucleoprotein A1; XIAP, X-linked inhibitor of apoptosis protein; PTEN, Phosphatase and tensin homolog; EMT, Epithelial to Mesenchymal Transition; Kras, Kirsten rat sarcoma virus (proto-oncogene); NF-κB, Nuclear factor kappa B; FOXC2, Forkhead box protein C2; **↑**, increased expression/activity; **↓**, decreased expression/activity.

### Ovarian Cancer

Resveratrol’s anti-tumor activity has also been reported in the context of ovarian cancer (OC) both *in vitro* and *in vivo*. Proposed mechanisms include autophagocytosis (Opipari et al., 2004), inhibition of proliferation and apoptosis induction (Liu et al., 2018), inhibition of glucose metabolism, and combined induction of autophagy and apoptosis (Tan et al., 2016). A crucial role in OC development is also played by several miRNAs (Alshamrani, 2020). For instance, the miR-34 family was found to be downregulated in OC tissues (Welponer et al., 2020) and one of its three members, miR-34a, was reported to function as a tumor suppressor in OC (Lv et al., 2018). A recent study showed a significant increase in miR-34a expression in resveratrol-treated OC cells (Yao et al., 2021), with consequent enhanced anti-tumor activity. The latter was attenuated by miR-34a-knockdown (Yao et al., 2021). This study also reported that miR-34a-mediated suppression of Bcl-2 in OC cells was responsible for the anti-cancer effects of resveratrol (Yao et al., 2021). Further confirmation of the resveratrol anti-neoplastic activity exerted via the ncRNAs-associated epigenetic modulation of intracellular pathways governing homeostasis, proliferation, death, and motility in OC cells has been provided by Vallino et al. (Vallino et al., 2020). In their study, combining miRNAs and lncRNAs microarray profiling of OC cells exposed to 24-hour resveratrol treatment with literature-based biochemical pathways and functional processes potentially associated with OC, resveratrol up-regulated seven miRNAs (including miR-1207-5p, miR-3665 and miR-4281) and five lncRNAs (including GAS5 and NBR2) and down-regulated two miRNAs and ten lncRNAs (including LINC00092, H19, MALAT1) (Vallino et al., 2020). See summary in Table 4.

### Prostate Cancer

Prostate cancer (PC) is one of the leading causes of cancer death among men of all races (Crawford, 2003). Genetic modifications in oncogenes and tumor suppressor genes, mutations in androgen receptors, and alterations in lncRNAs or miRNAs are some of the risk factors associated with PC development (Marcelli et al., 2000; Taylor et al., 2010; Weiss et al., 2014). Pro-inflammatory cytokines, such as IL-6, are also critical for the progression of PC as they are associated with increased activation of the oncogene signal transducer and activator of transcription 3 (STAT3) (Culig et al., 2005) (Rojas et al., 2011). The interplay between lncRNAs and miRNAs has also been reported as key to modulate the expression of genes involved in PC development and progression (Hu et al., 2022). lncRNAs such as PC associated transcript-1 and -3 (PCAT1 and PCAT3) increase proliferation of cancer cells whereas PCAT29 decreases both proliferation and migration of PC cells (Malik et al., 2014). A recent study reported that both gene and protein level expression of PCAT29 were reduced in tumors compared to normal prostate tissues (Al Aameri et al., 2017). The IL-6/STAT3/miR-21 signaling pathway mediated this reduction, and IL-6 was the main inducer of PCAT29 suppression through STAT3 activation. STAT3 then activated the downstream target miR-21, which in turn downregulated PCAT29. By contrast, inhibition of miR-21 restored the basal expression of PCAT29 (Al Aameri et al., 2017). Notably, resveratrol was able to reverse PCAT29 downregulation via abrogation of IL-6/STAT3/miR-21 signaling, and a direct targeting of miR-21 by resveratrol was also suggested (Al Aameri et al., 2017). Other studies have similarly reported the ability of resveratrol to alter PC miRNA profiles. For instance, Dhar et al. (Dhar et al., 2015) showed that both resveratrol and pterostilbene, by repressing several members of the oncogenic miR-17 family, were able to rescue the defective expression of PTEN in prostate cancer. These miRNAs are indeed overexpressed in PC and directly target the 3′UTR of tumor suppressor gene PTEN (Phosphatase and Tensin homolog), reducing its expression (Dhar et al., 2015). Furthermore, Chen et al. (Chen et al., 2021) reported the downregulation of lncRNA DIO3OS by resveratrol in benign prostatic hyperplasia (BPH). Although the pathophysiological role of DIO3OS remains poorly known, Chen et al. demonstrated that DIO3OS is highly expressed in BPH tissues. Within this environment, acting via miR-656-3p and miR-485-5p, DIO3OS promotes epithelial-mesenchymal transition (EMT) by BPH-1 cells through the upregulation of two EMT key effectors, CTGF and ZEB1 (Chen et al., 2021). Moreover, both TGF-β1 and resveratrol modulated DIO3OS in a Smads-dependent manner: TGF-β1 upregulated DIO3OS expression, which favored EMT, whereas resveratrol downregulated it, suppressing EMT (Chen et al., 2021). See summary in Table 4.

### Other Cancers

Colorectal cancer (CRC) is the third most common cancer worldwide. Mutations in the oncogene Kras are the most prevalent CRC drivers (Haggar and Boushey, 2009). An *in vivo* study showed the effects of a resveratrol-supplemented diet on genetically engineered mice, where the Kras locus is activated specifically in the distal colon (Saud et al., 2014). The intervention resulted in a 60% inhibition of tumor production before tumors were visible with colonoscopy. In animals with established tumors, 33% exhibited complete remission whereas 97% showed a decrease in tumor size (Saud et al., 2014). Analysis of miRNA expression in both tumor and non-tumor tissue of resveratrol fed mice indicated an increased expression of miR-96, suggesting that resveratrol may inhibit translation of Kras mRNA by inducing miR-96 overexpression (Saud et al., 2014). Using the human colorectal adenocarcinoma cell line HT-29, a recent *in vitro* study showed that resveratrol, alone or in combination with the telomerase inhibitor BIBR1532, reduced cell viability and decreased the expression of a set of CRC-associated lncRNAs, including CCAT1, CRNDE, HOTAIR, PCAT1, PVT1, and SNHG16 (Cesmeli et al., 2022). The study of Fu et al. (Fu J. et al., 2019) suggested that the effect of the resveratrol derivative, triacetyl resveratrol (TCRV), in inhibiting pancreatic cancer cells (AsPC-1) migration, invasion, and EMT was mediated by the miR-200 family members. The authors indirectly confirmed the hypothesis that the miR-200 family mediated the biological effects of TCRV as the overexpression of anti-miR-200a/b/c counteracted the inhibitory effects of TCRV on AsPC-1 cells migration and invasion (Fu J. et al., 2019). Besides, treatment with TCRV inhibited the activity of the miR-200 family’s transcriptional target, Zeb1, one of the main EMT inducers (Fu J. et al., 2019). In multiple myeloma (MM) cells, where the lncRNA NEAT1 is highly expressed and induces cells proliferation, migration and invasion, resveratrol reversed the negative effect of NEAT1 through the Wnt/β-catenin signaling pathway (Geng et al., 2018). The anti-tumor effects of resveratrol have been also reported in melanoma, both *in vitro* and *in vivo*, where this flavonoid significantly decreased the expression of the oncogenic miR-221 by regulating (NF-κB) (RELA) activity (Wu and Cui, 2017). In the context of lung cancer, a microarray analysis of gene expression reported by Yang et (Yang et al., 2015) indicated that lncRNA expression profiles were altered in resveratrol-treated lung cancer cells, with 21 upregulated and 19 downregulated. The lncRNA AK001796 displayed the most altered expression, which was high in untreated lung cancer cell lines but significantly low with resveratrol (Yang et al., 2015). In the same study, *in vitro* and *in vivo* experiments supported the proposition that AK001796 acts as an oncogene in lung cancer carcinogenesis and is involved in the anticancer effects of resveratrol (Yang et al., 2015). Another study investigating the epigenetic effects of resveratrol in lung cancer identified a signaling cascade, resveratrol|miRNA-520h|PP2A/C|Akt|NF-κB|FOXC2, in which resveratrol inhibits both gene and protein expression of the transcriptional factor FOXC2 by downregulating the oncogenic miRNA-520h and the downstream signaling cascade (Yu et al., 2013). FOXC2 is an inducer of EMT, tumor angiogenesis and metastasis, and elevated levels of FOXC2 are associated with advanced cancer and poor prognosis (Yu et al., 2013). Interestingly, treatment with resveratrol reversed the effect of FOXC2 and induced MET (transformation of mesenchyme to epithelium), the opposite of EMT (Yu et al., 2013). See summary in Table 4.

## Conclusion

**FIGURE 1:**
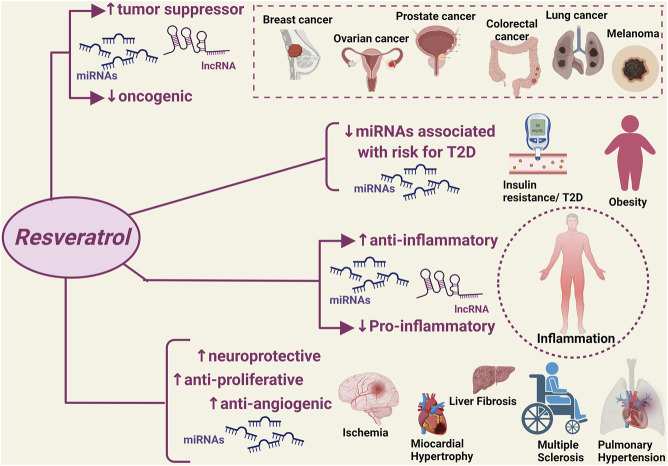
Modulatory effect of resveratrol on disease-associated non-coding RNAs. miRNAs, micro RNA; lncRNA, non-coding RNAs. ↑: increased expression/activity; ↓: decreased expression/activity.

A significant number of studies published over the last 5 years has provided robust evidence that the natural flavonoid resveratrol can modulate critical homeostatic processes by interacting with a wide range of ncRNAs in the context of disease states that are typically characterized by a high inflammatory burden and oxidative stress (Figure 1). The discovery of such resveratrol-ncRNAs interactions adds to the well-known anti-inflammatory and antioxidant effects of this natural compound by allowing the identification of a series of mediators that could serve as additional druggable targets as well as markers of disease onset and progression. Notably, the reported modulatory effects of resveratrol on ncRNAs also applies to disease states with limited available therapeutic options, i.e., ovarian cancer, liver fibrosis and failure, and pulmonary hypertension, providing much-needed information for the discovery of new therapeutic strategies. Furthermore, the resveratrol-ncRNAs interactions seem to also exert beneficial effects that go beyond the established anti-inflammatory and antioxidant effects of resveratrol, e.g., addressing drug resistance in cancer through the increased sensitivity of cancer cells to conventional chemotherapeutic regimens.

Despite the promising results of the studies discussed in this review, additional research is urgently warranted to confirm the potential therapeutic effects of resveratrol and its analogues in robust animal models of disease using a wide range of doses, routes of administration, and treatment duration, and possible combinations with conventional medications and/or other natural compounds. This is particularly important given the complex pharmacokinetics of resveratrol and the delicate balance between efficacy and toxicity with this flavonoid (Salehi et al., 2018; Shaito et al., 2020), necessitating the identification of the most appropriate serum and tissue concentration ranges. Only then can the assessment of the potential therapeutic benefits of the resveratrol-ncRNAs interaction be conducted in patient cohorts using appropriately designed trials.
